# Commentary: Molecular and immunological features of TREM1 and its emergence as a prognostic indicator in glioma

**DOI:** 10.3389/fimmu.2025.1577773

**Published:** 2025-06-17

**Authors:** Jihao Xue, Zhilin Luo, Tao Wang, Qijia Yin, Ligang Chen

**Affiliations:** ^1^ Department of Neurosurgery, the Affliliated Hospital, Southwest Medical University, Luzhou, Sichuan, China; ^2^ Department of Geriatric Medicine, The Affliliated Hospital, Southwest Medical University, Luzhou, Sichuan, China; ^3^ Department of Urology or Nursing, Sichuan Provincial People's Hospital East Sichuan Hospital & Dazhou First People’s Hospital, Dazhou, Sichuan, China

**Keywords:** proportional hazards (PH) assumption, glioma, Cox regression, nomogram, prediction

We read with great interest the recently reported study published in *Front. Immunol.* by Zhang et al. (1). The researchers employed both univariate and multivariate Cox regression analysis to validate TREM1 as an independent prognostic biomarker for gliomas. Following this, they developed a nomogram utilizing the TREM1 expression level, WHO grade, gender, age, radiotherapy, chemotherapy, and IDH status, sourced from the TCGA cohort, to forecast the 1-year, 3-year, and 5-year survival probabilities of glioma patients. This study demonstrated that the nomogram possessed satisfactory predictive capability. Acknowledging the significant contributions of this study, we have identified certain deviations in the authors’ application of the Cox proportional hazards (CoxPH) model that are still unstated and unresolved.

Mixed censoring results (i.e., interval-censoring and right-censoring occurrences) could arise from the established criteria (2). Interval censoring may occur if the end event of glioma identified by medical records takes place in between follow-ups. Right censoring may occur if the diagnosis is made between the conclusion of the follow-up and the data analysis period. Dealing with right-censoring data is the main emphasis of the CoxPH model. The accelerated failure time (AFT) model, on the other hand, is usually chosen for situations that involve an extensive variety of censored data types (3). By adequately modifying the likelihood function, the AFT model may effectively handle data that has been left, right, or interval censored (4). The R packages (‘icenReg’ and ‘survival’) are useful for fitting and analyzing mixed censored data as well as estimating event timings (5).

Furthermore, from the standpoint of modeling strategy, the CoxPH model assumes that the hazard ratio is constant over the course of the follow-up period, meaning that the influence of covariates does not change over time (6). Inaccurate prediction findings and skewed statistical conclusions drawn from the model may result from breaking the proportional hazards (PH) assumption (7, 8). However, a number of factors may contribute to the frequent emergence of nonproportionality of hazards in practice (9). Schoenfeld residuals or other alternative methods should be used by the investigators to evaluate the PH assumption of the association between covariates and outcomes (10). If there is a consistent pattern of change over time in the residuals, it suggests that the covariate’s effect may fluctuate over time. Instead of using the conventional Cox proportional hazards model, writers should use the Cox model including time-varying effects, the stratified Cox model, or the AFT model when the proportional hazards assumption fails to be met (11–13).

**Figure 1:**
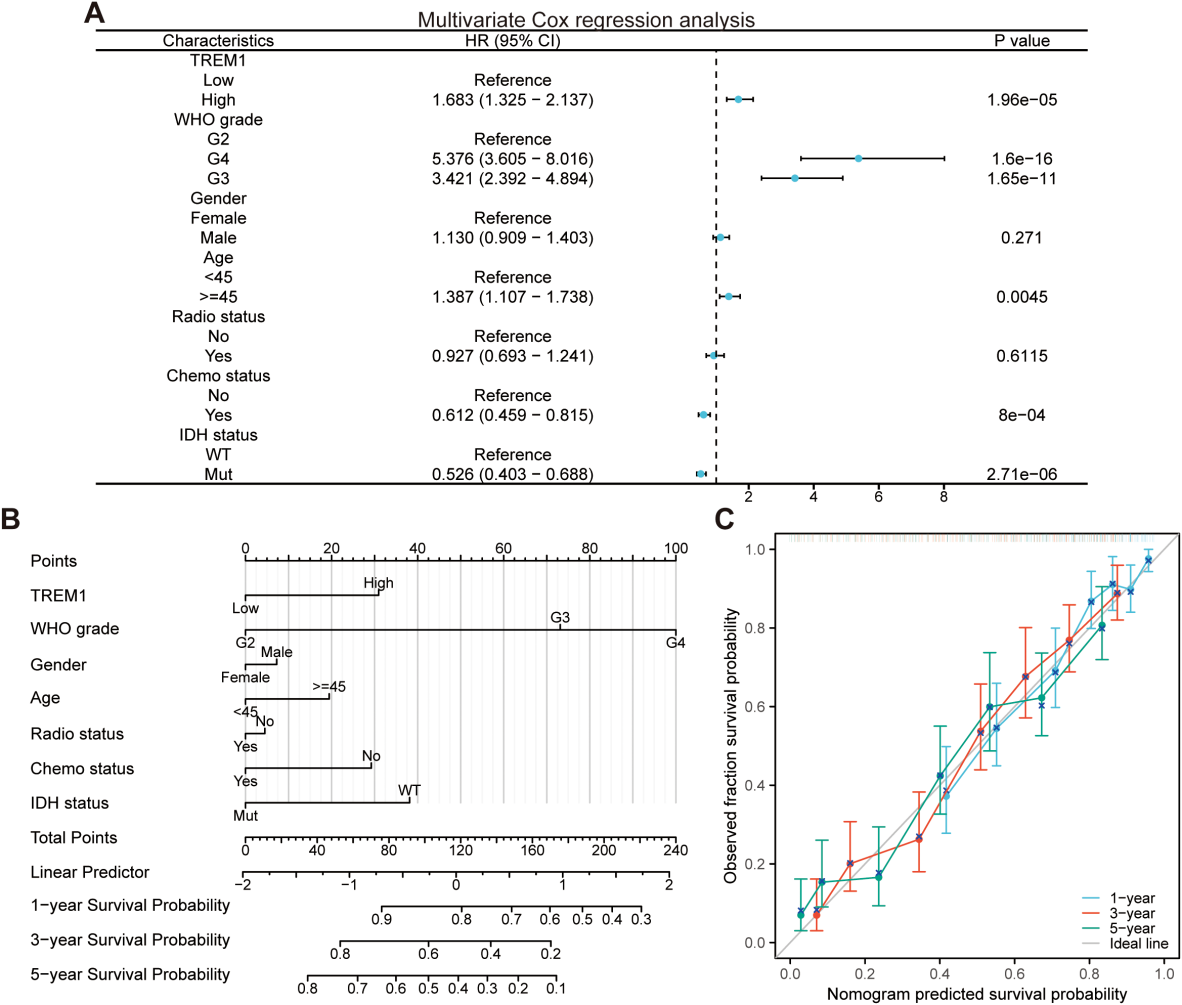
Development and evaluation of the nomogram. **(A)** Multivariate Cox regression analysis in the TCGA cohort. **(B)** Nomogram for predicting the survival probability of glioma patients. **(C)** Calibration curves for predicting the 1-year, 3-year, and 5-year survival probability of glioma patients.

Similarly, we performed the univariate and multivariate Cox regression analysis (**Figure 1A**) of relevant clinical parameters as conducted by Zhang et al. (1) on glioma patients from the TCGA cohort, and verified the PH assumption for the multivariate Cox regression model (**Table 1**). Afterwards, we built a nomogram (**Figure 1B**) utilizing multivariate Cox regression results and plotted calibration curves (**Figure 1C**) to demonstrate the predictive value of the nomogram in prognosis. The findings indicated that the global test failed to meet the PH assumption, potentially undermining the statistical credibility and precision of the predictive model introduced by Zhang et al. (1) (**Table 1**).

**Table 1:** PH assumption test to multivariate Cox regression analysis.

Variable	Chi-Square value	Degree of freedom	*P* value
TREM1	1.184	1	0.277
WHO grade	11.342	2	0.003
Gender	2.877	1	0.090
Age	1.384	1	0.240
Radiotherapy status	9.444	1	0.002
Chemotherapy status	0.104	1	0.747
IDH status	17.229	1	3.31e-05
Global	34.465	8	3.35e-05

If the *p*-value in the global test > 0.05, it indicates that the multivariate Cox regression adheres to the PH assumption.

As a predictive model based on statistical principles, the accuracy of nomograms largely depends on the richness and diversity of the training data. Nonetheless, the sample size incorporated in the development of the nomograms within this research is comparatively modest, thereby fundamentally limiting the predictive precision and the capacity for generalization of the models in question. Furthermore, they did not utilize an external validation dataset to evaluate the predictive performance of the nomogram model subsequent to its development, thereby failing to demonstrate the applicability of the model across different patient populations. To address these challenges, future studies should strive to collect more extensive and representative multicenter datasets for the development of nomogram models. Concurrently, after the construction of the model, a rigorous validation using independent external cohorts should be carried out to comprehensively assess the predictive performance of the model.

In conclusion, taking into account the proportionate hazards assumption and the possible ramifications of censoring events, we conclude that a reevaluation is necessary. Despite our concerted efforts to underscore the prerequisites for utilizing multivariate Cox regression model and developing nomogram, numerous publications continue to overlook the PH assumption in their investigations. This underscores the fact that the meticulous validation of models remains an overlooked yet indispensable component within the realm of scientific inquiry. Therefore, we strongly recommend that the scientific community adopts strict and standardized methods when constructing predictive models, and ensures compliance with the prerequisites for the appropriate use of these models.
